# Chronotype and self-reported sleep, alertness, and mental health in U.S. sailors

**DOI:** 10.1186/s40779-021-00335-2

**Published:** 2021-08-10

**Authors:** Elizabeth M. Harrison, Alexandra P. Easterling, Emily A. Schmied, Suzanne L. Hurtado, Gena L. Glickman

**Affiliations:** 1 Center for Circadian Biology, University of California San Diego, San Diego, USA; 2 Leidos, Inc., San Diego, USA; 3 Health and Behavioral Sciences Department, Naval Health Research Center, San Diego, USA; 4 School of Public Health, San Diego State University, San Diego, USA; 5 Departments of Psychiatry and Neuroscience, Uniformed Services University of the Health Sciences, Bethesda, USA

**Keywords:** Circadian, Chronotype, Sleep, Military, Readiness, Depression, Anxiety, Post-traumatic stress disorder (PTSD)

## Abstract

Service members are at risk for sleep and psychological conditions affecting their readiness. Chronotype (“morningness” or “eveningness”) is strongly associated with sleep, health and performance. The objective of this study was to examine associations between validated measures of chronotype and sleep quality, daytime functioning, alertness, and symptoms of depression, anxiety, and post-traumatic stress disorder (PTSD) in US service members (*n* = 298). Although predominantly young males (who skew toward eveningness in civilian populations), these Sailors skewed toward morningness (35.6% morning, 51.3% intermediate). Eveningness was associated with symptoms of depression and post-traumatic stress disorder (PTSD) (*P* < 0.01), less time in bed (*P* < 0.05), more sleep disruption (*P* < 0.01), and poorer daytime functioning and alertness (*P* < 0.05). Evening types were less likely to consider sleep important for performance (*P* < 0.05). To maximize service member readiness, schedules should be aligned with endogenous rhythms, whenever possible, and evening chronotypes may benefit from targeted interventions. Chronotype should be examined alongside health and readiness in service members.

**Supplementary Information:**

The online version contains supplementary material available at 10.1186/s40779-021-00335-2.

**Dear Editor,**

Sleep is regulated in part by internal, self-sustaining daily (“circadian”) rhythms. While sleep and circadian rhythms play a critical role in service member readiness, disturbances of these important physiological processes are unfortunately common in military populations, which face unique challenges [1]. “Chronotype” is a circadian phenotype, with both biological and environmental underpinnings, that describes one’s alignment with the solar cycle and either a relatively early (morningness) or late (eveningness) schedule preference. In civilians, these preferences correlate with physiological measures of internal timing, such as the nocturnal rise in melatonin [2].

Chronotype is generally normally distributed in civilians and has been linked to both sex and age, with males and younger adults skewing toward eveningness [2]. There is also a relationship between chronotype and mental health outcomes [3]. High rates of depression, anxiety, and post-traumatic stress disorder (PTSD) have been observed in military populations [4]; yet, research on chronotypes in service members is limited.

Sailors from three ships who were recruited to participate in a circadian health intervention (to be published separately) were administered questionnaires on sleep, alertness and mental health. Items included questions on demographics and the reduced morningness-eveningness questionnaire (rMEQ) [5], which yields both continuous and categorical measures of chronotype (definitely morning, moderately morning, neither type, moderately evening, or definitely evening; this can be further binned into morning, evening and neither types). Average sleep duration (Pittsburgh sleep quality index, PSQI) and recent symptoms of depression, anxiety, posttraumatic stress, and sleep disturbances were assessed using validated scales [Generalized anxiety disorder scale (GAD-7), 8-item version of the patient health questionnaire (PHQ-8), Posttraumatic stress disorder checklist version 5 (PCL-5), and PSQI, respectively; higher scores = greater symptomatology on all scales]. An additional four items measured readiness over the past month, and two asked about perceived importance of sleep for performance. Participants reporting a currently diagnosed sleep disorder were excluded from participation. Chronotype was examined for relationships with mental health symptoms, sleep, and alertness using chi-squared analyses, correlations, and ANOVA, as appropriate (GraphPad Prism; La Jolla, CA and SPSS, IBM; Armonk, NY). The study protocol was approved by the Institutional Review Board of the Naval Health Research Center in compliance with all applicable federal regulations governing the protection of human subjects. All participants consented to participate.

The demographics are shown in Additional file 1: Table S1 (*n* = 298). The majority of the sample were males (82.9%) between the ages of 17–29 (65.4%). Despite being mostly younger males, this sample was skewed toward morningness (35.6% morning, 51.3% intermediate) and earlier relative to normative data (Additional file 1: Fig. S1). The skewness observed herein was not totally unexpected in this unique population. Possible explanations include military-specific environmental cues (early rise times) or self-selection of military careers by morning types. Chronotype should be examined across individual military careers to assess the trajectory of age-related changes compared with civilians.

Sleep, alertness, and readiness parameters are reported in Additional file 1: Table S2. The rates of reported accidents or near misses did not vary by chronotype (*χ*^2^_(2)_ = 1.0, *P* = 0.61 and *χ*^2^_(2)_ = 2.0, *P* = 0.37, respectively), although the rates for both were low overall. The frequency of struggling to stay awake and falling asleep on duty varied by chronotype (*χ*^2^_(8)_ = 17.22, *P* < 0.05 and *χ*^2^_(8)_ = 18.53, *P* < 0.05, respectively). Morning types reported struggling to stay awake and falling asleep on duty less frequently than evening types; however, evening types were less likely to think sleep was important for performance (Additional file 1: Table S2). Future efforts might consider interventions to benefit evening types and/or younger service members, such as morning light administration and targeted sleep and circadian educational programs.

The average reported sleep duration for the sample was about 5 h and 40 min, and the average calculated time spent in bed, based on the reported typical wake- and bedtimes, was about 6 h and 30 min (Additional file 1: Table S2). Time spent in bed, but not time asleep, varied by chronotype (*F*_(2,294)_ = 3.57, *P* < 0.05 and *F*_(2,295)_ = 1.59, *P* = 0.21, respectively), as did bedtimes (*F*_(2,294)_ = 10.98; *P* < 0.001) and waketimes (*F*_(2,294)_ = 3.35; *P* < 0.05). Morning types spent about 40 min longer in bed than evening types (Additional file 1: Table S2) and went to bed about 30 and 60 min earlier than intermediate and evening types, respectively. Early types also woke about 30 min earlier than intermediate types (Additional file 1: Table S2). Early start times are among several military-specific issues that affect alertness and sleep [1]. Modest shifts in schedules, where possible, may better align with peak performance in evening types. Despite differences in the duration of time in bed by chronotype, sleep efficiency (%), a measure of sleep quality derived from the reported sleep duration and calculated time in bed, did not vary by chronotype (*P* = 0.69).

Correlations and associated statistics for relationships among chronotype, sleep quality, and mental health are shown in Additional file 1: Table S3. Lower values on the rMEQ scale indicate eveningness; thus, a negative correlation with morningness is equivalent to a positive correlation with eveningness. Morningness was negatively correlated with symptoms of depression and PTSD but not anxiety. Additionally, morningness was negatively correlated with global sleep disruption, daytime sleepiness, poorer sleep quality, and sleep latency. Thus, chronotype may be an identifiable risk factor, or potential mitigating factor, for some of the known difficulties encountered by service members.

Chronotype is an understudied facet of circadian rhythm research in military populations that could provide a mechanism for performance optimization. Service members are at increased risk for mental health and sleep issues, [1, 4] both of which were related to chronotype in these unadjusted analyses. Future studies should consider examining chronotype as a risk factor for mental health and sleep outcomes.

## Supplementary Information

**Additional file 1.Table S1**: Participant characteristics. **Table S2**: Sleep, alertness, and readiness by chronotype. **Table S3**: Correlations between chronotype, sleep, and mental health outcomes. **Figure S1**: Chronotype by age and compared to normative data.

## Data Availability

The datasets generated and/or analyzed during the current study are not publicly available because they are the property of the United States Government but may be available from the corresponding author on reasonable request.

## Abbreviations

GAD-7: generalized anxiety disorder scale
rMEQ: morningness–eveningness questionnaire-reduced version
PCL-5: posttraumatic stress disorder checklist version 5
PHQ-8: 8-item version of the patient health questionnaire
PSQI: Pittsburgh sleep quality index
PTSD: post-traumatic stress disorder
