# Supplementary material for: Chronotype and self-reported sleep, alertness, and mental health in U.S. sailors
Source: Mil Med Res. 2021 Aug 10;8:43. doi: 10.1186/s40779-021-00335-2 (PMC8353852; doi:10.1186/s40779-021-00335-2)
Supplement: Supplementary file 1 — Additional file 1.Table S1: Participant characteristics. Table S2: Sleep, alertness, and readiness by chronotype. Table S3: Correlations between chronotype, sleep, and mental health outcomes. Figure S1: Chronotype by age and compared to normative data. [file 40779_2021_335_MOESM1_ESM.pdf]

Table S1 Participant characteristics

Item	*n* (%)
Gender	
Male	247 (82.9)
Female	51 (17.1)
Age	
17-24	119 (39.9)
25-29	76 (25.5)
30-39	91 (30.5)
40+	12 (4.0)
Race/Ethnicity	
White	124 (41.6)
Black	47 (15.8)
Hispanic	51 (17.1)
Asian	36 (12.1)
Other/ Multiple	40 (13.4)
Military Component	
Active regular	293 (98.3)
Active reserve	5 (1.7)
Paygrade/Rank	
E1-E4	113 (37.9)
E5-E6	126 (42.3)
E7-E9	29 (9.7)
O1-O3	29 (9.7)
O4 and higher	1 (0.3)
Occupation/Department	
Engineering	81 (27.2)
Ship operations	66 (22.2)
Ordnance/Warfare systems	30 (10.1)
Communications/Information systems	28 (9.4)
Supply/Logistics	25 (8.4)
Electronics	23 (7.7)
Other	44 (14.8)

E enlisted, *O* officer

Table S2 Sleep, alertness, and readiness by chronotype

Item		Morning	Intermediate	Evening	*P value*
Chronotype (%)	*Overall*	35.6	51.3	13.1	
	Moderately	31.2	--	12.8	
	Definitely	4.4	--	0.3	
Struggle to stay awake (%)	*Never*	26.4	25.0	20.5*	*P* < 0.05
	A few times	59.4	53.9	43.6	
	Several times/week	10.4	13.2	12.8	
	> half the days	1.9	4.6	17.9	
	Nearly everyday	1.9	3.3	5.1	
Fall asleep during duty (%)	*Never*	77.4	66.4	59.0*	*P* < 0.05
	A few times	20.6	27.0	23.1	
	Several times/week	0.9	2.6	5.1	
	> half the days	0.0	2.0	10.3	
	Nearly everyday	0.9	2.0	2.6	
Accident (%)	*Yes*	6.7	9.2	5.1	*P* = 0.61
	No	93.3	90.8	94.9	
Near miss (%)	*Yes*	10.5	15.9	17.9	*P* = 0.37
	No	89.5	84.1	82.1	
Importance of sleep for health (%)	*Not at all*	0.0	0.0	0.0	*P* = 0.37
	Slightly	1.3	1.5	6.1	
	Moderately	13.2	12.8	21.2	
	Very	42.1	48.9	33.3	
	Extremely	43.4	36.8	39.4	
Importance of sleep for performance (%)	*Not at all*	1.3	0.0	3.0^	*P* < 0.05
	Slightly	1.3	0.8	9.1	
	Moderately	10.5	12.0	21.2	
	Very	34.2	43.6	27.3	
	Extremely	52.6	43.6	39.4	
Sleep (h)	*Bedtime*	21.85(1.23)^	22.40(1.28)	22.83(1.05)*	*P* < 0.001
	Waketime	4.53(0.88)^	4.97(1.67)	4.87(1.00)	*P* < 0.05
	Sleep	5.82(1.35)	5.60(1.23)	5.42(1.08)	*P* = 0.21
	Time in bed	6.67(1.30)	6.48(1.32)	6.02(1.22)*	*P* < 0.05

An asterisk (*) denotes that post-hoc mean value is statistically different from Morning types for a given parameter, and a carrot (^) indicates a difference from Neither types. For variables with multiple responses, asterisks from comparisons across chronotype group are shown on the first row in the table (e.g., *Never* for struggling to stay awake). Sample sizes vary (n = 242-298), and effect sizes range from small to large (all Cohen's d's > 0.37, all Cramer's V's > 0.15). Mean (± SD) bed- and wake-times for the entire sample were 22.27 ± 1.57 and 4.80 ± 1.37, respectively. Average sleep duration (mean ± SD) for the sample was 5.65 ± 1.27 hours, and average time in bed was 6.48 ± 1.32 hours.

Table S3 Correlations between chronotype, sleep and mental health outcomes

Item	PHQ-8	GAD-7	PCL-5	PSQI: Global	PSQI: Daytime dysfunction
rMEQ	-0.17**	-0.10	-0.15**	-0.17**	-0.23***
PHQ-8	--	0.73***	0.78***	0.46***	0.55***
GAD-7		--	0.80***	0.39***	0.50***
PCL-5			--	0.43***	0.51***
PSQI: Global				--	0.47***

Lower values on the rMEQ scale indicate eveningness; thus, a negative correlation with morningness is equivalent to a positive correlation with eveningness. Morningness was negatively correlated with greater symptomology in terms of depression, PTSD, and sleep impairment, including global sleep disruption and daytime sleepiness (PSQI). Additionally, greater morningness was negatively correlated with poorer sleep quality (PSQI; r = -0.23, P < 0.001) and a longer sleep latency (time to fall asleep) (PSQI; r = -0.19, P < 0.01). *rMEQ* morningness-eveningness questionnaire-reduced version; *GAD-7* generalized anxiety disorder scale; *PCL-5* posttraumatic stress disorder checklist version 5; *PHQ-8* 8-item version of the patient health questionnaire, depression; *PSQI* Pittsburgh sleep quality index; *PTSD* post-traumatic stress disorder

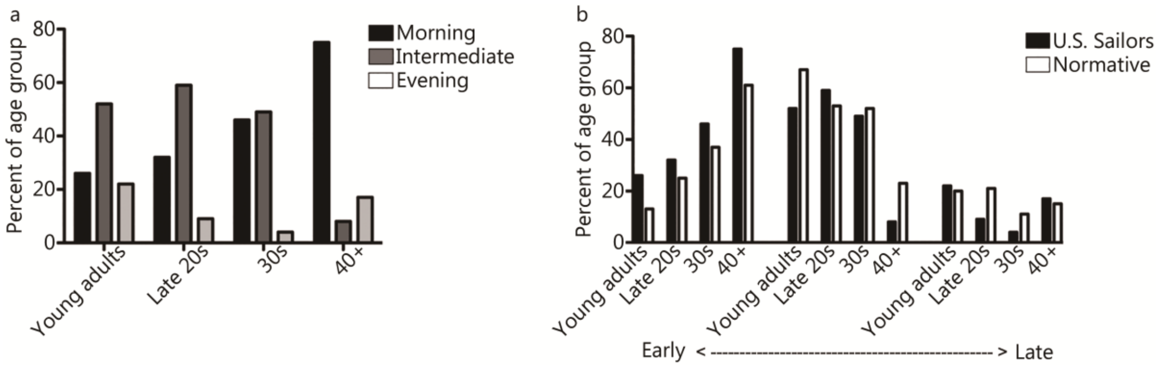

Fig. S1 Chronotype by age and compared to normative data. a, depicts the percent of each age group who were categorized as early, late, and intermediate types. The age item on the questionnaire was divided into four bins: young adults (17-24), late 20s (25-29), 30s (30-39), and 40+ (40 or above). The distribution skews towards morningness generally. b, plots the data in the same age groups relative to the normative data from Adan and Almirall, 1991. Chronotype did not vary by gender ($X^2_{(2)} = 3.02$, $P = 0.22$) but the proportion of morning types increased with age ($X^2_{(6)} = 30.02$, $P < 0.001$) and rank ($X^2_{(6)} = 24.85$, $P < 0.001$). Post-hoc analyses revealed significant differences in chronotype distribution for all age group comparisons ($P < 0.05$ for 17-24 vs. 25-29, all other $P < 0.001$), with the exception of 25-29 vs. 30-39 ($P = 0.06$). For comparison to normative data, chronotype distribution within each age group was analyzed. On the questionnaire, age was binned into four categories: 17-24, 25-29, 30-39, and 40+, which were relatively comparable to those in the normative data (17-23, 24-30, 31-39, and 41-50). Those comparable age bins will be called "young adults," "late 20s," "30s," and "40+," respectively, throughout this report. Further, though the two samples come from different countries (e.g., individuals in Spain have later bedtimes than those in the U.S.), the rMEQ scoring accounts for such differences by having the range for scoring for each item adjusted by region, so that overall scores can be comparable across different populations. Chronotype was binned into three categories as in Natale et al.[1] - evening, intermediate, and morning types. There were more morning types in our sample of Sailors than in normative data across all age groups (Fig. S1b; $X^2_{(6)} = 30.02$, $P < 0.001$). Post-hoc tests revealed significantly more morning types amongst the youngest two groups ($P < 0.01$ for the young adults and $P < 0.05$ for the late 20s), but not for the two older groups (both $P > 0.09$). As might be expected based on age results, morningness was significantly associated with years in service ($r = 0.18$, $P < 0.01$), but there was no independent relationship between years in service and morningness after controlling for age ($r = 0.01$, $P = 0.83$).

[1] Natale V, Esposito MJ, Martoni M, Fabbri M. Validity of the reduced version of the morningness-eveningness questionnaire. Sleep Biol Rhythms. 2006; 4(1):72-4.
